# A qPCR Assay for the Quantification of Selected Genotypic Variants of Spodoptera frugiperda Multiple Nucleopolyhedrovirus (*Baculoviridae*)

**DOI:** 10.3390/v16060881

**Published:** 2024-05-30

**Authors:** Cindy S. Molina-Ruiz, Jesús Alejandro Zamora-Briseño, Oihane Simón, Rodrigo Lasa, Trevor Williams

**Affiliations:** 1 Instituto de Ecología AC (INECOL), Xalapa, Veracruz 91073, Mexico; cindymolinarz@gmail.com (C.S.M.-R.); alejandro.zamora@inecol.mx (J.A.Z.-B.); rodrigo.lasa@inecol.mx (R.L.); 2 Institute for Multidisciplinary Research in Applied Biology, Universidad Pública de Navarra, 31006 Pamplona, Spain; oihane.simon@unavarra.es

**Keywords:** alphabaculovirus, fall armyworm, genotypic diversity, efficiency, copy number determination

## Abstract

Alphabaculoviruses are lethal dsDNA viruses of Lepidoptera that have high genetic diversity and are transmitted in aggregates within proteinaceous occlusion bodies. This mode of transmission has implications for their efficacy as biological insecticides. A Nicaraguan isolate of Spodoptera frugiperda multiple nucleopolyhedrovirus (SfMNPV-NIC) comprising nine genotypic variants has been the subject of considerable study due to the influence of variant interactions on the insecticidal properties of mixed-variant occlusion bodies. As part of a systematic study on the replication and transmission of variant mixtures, a tool for the accurate quantification of a selection of genotypic variants was developed based on the quantitative PCR technique (qPCR). First, primer pairs were designed around a region of high variability in four variants named SfNic-A, SfNic-B, SfNic-C and SfNic-E to produce amplicons of 103–150 bp. Then, using cloned purified amplicons as standards, amplification was demonstrated over a dynamic range of 10^8^–10^1^ copies of each target. The assay was efficient (mean ± SD: 98.5 ± 0.8%), reproducible, as shown by low inter- and intra-assay coefficients of variation (<5%), and specific to the target variants (99.7–100% specificity across variants). The quantification method was validated on mixtures of genotype-specific amplicons and demonstrated accurate quantification. Finally, mixtures of the four variants were quantified based on mixtures of budded virions and mixtures of DNA extracted from occlusion-derived virions. In both cases, mixed-variant preparations compared favorably to total viral genome numbers by quantification of the *polyhedrin* (*polh*) gene that is present in all variants. This technique should prove invaluable in elucidating the influence of variant diversity on the transmission and insecticidal characteristics of this pathogen.

## 1. Introduction

Alphabaculoviruses are double-stranded DNA viruses in the family *Baculoviridae* [1]. These viruses are known for their ability to form proteinaceous occlusion bodies (OBs) that occlude groups of occlusion-derived virions (ODVs), each of which comprise one or several rod-shaped nucleocapsids surrounding each viral genome [2,3]. Alphabaculoviruses infect lepidopterans and are widely employed as biological insecticides due to their high pathogenicity and host specificity [4].

Horizontal transmission occurs when lepidopteran larvae become infected after ingesting foliage contaminated with OBs [5]. In the midgut, alkaline conditions and proteolytic enzymes degrade the OBs, releasing the ODVs. Infection occurs in two stages. During primary infection, the ODVs infect the midgut epithelial cells. Then, budded virions (BVs) released from the basal membrane of the primary infected cells are responsible for secondary infection of the insect during which BVs are disseminated through the hemolymph and the tracheal system to the cells of other tissues. Late in infection, ODVs are retained in the nucleus and are occluded into progeny OBs. Days later, infected caterpillars die, and an enzymatic liquefaction of their bodies releases millions of OBs into the environment for the following cycle of transmission [2].

Nucleopolyhedrovirus OBs are an example of collective infectious units as they package multiple genomes in a single viral structure [6]. Forming these aggregates can result in more efficient infections since a single virion has a low probability of establishing an infection [7,8]. Furthermore, the genomes present in each OB can correspond to different genotypic variants and can provide a mechanism for amelioration of the genetic bottleneck that occurs during horizontal transmission [9]. Increased diversity in the founder populations that infect insects may provide advantages in overcoming heterogeneity in cellular susceptibility to infection and the host’s immune response [10,11,12].

Alphabaculovirus genomes have a high degree of structural diversity [13], which includes indels, natural recombinants, transposable elements and single nucleotide polymorphisms (SNPs) that generate marked genotypic diversity within each natural isolate [14,15]. Natural isolates may also include genomes with significant deletions and defective variants that survive by complementation with complete genotypes in co-infected cells [16,17,18]. These individual variants often differ markedly in their insecticidal characteristics, including OB pathogenicity, speed of kill and OB production traits [19,20,21,22].

An isolate of Spodoptera frugiperda multiple nucleopolyhedrovirus (species name: *Alphabaculovirus spofrugiperdae*), originally isolated in Nicaragua (SfMNPV-NIC) [23,24], comprises at least nine genotypic variants isolated by plaque purification in Sf9 cells and that vary in restriction endonuclease profiles [25] and insecticidal phenotype [26]. Defective variants are also present in the population; they affect transmissibility and survive by complementation [16]. In fact, in SfMNPV, none of the individual variants are as pathogenic [27] or productive [28] as the natural isolate, implying that interactions among variants are a key component of virus fitness.

To better comprehend how host–variant and variant–variant interactions impact the transmission and replication dynamics of variant populations, a variant-specific virus quantification method is essential. In the present study, four genotypic variants were selected that differed in their phenotypic traits and in their prevalence in the SfMNPV-NIC isolate. Previous studies involving plaque purification and semi-quantitative PCR amplicon densitometric analyses found that variant B (SfNic-B) was the most abundant variant in the population, representing ~60% of variants [16,25]. SfNic-B comprises the complete genome of the virus (133 kb) [24]. Three other variants, SfNic-A, SfNic-C and SfNic-E, present important deletions of 10.3, 16.4 and 13.7 kb, respectively, in an auxiliary gene-rich region located between map units 14.8 and 27.6 [26]. SfNic-A and SfNic-E were reported to occur at low prevalence (~1–3%) in the natural isolate, whereas SfNic-C was more prevalent (~30%) despite its large deletion. These variants also differ phenotypically. SfNic-A has a higher OB production than the natural isolate, whereas SfNic-E has the slowest speed of kill and low OB pathogenicity [16,25]. In contrast, SfNic-C is defective for transmission because it lacks the *pif*1 and *pif*2 genes that are essential for infection of midgut cells [29,30]. It can only persist in the population by coinfection and complementation with PIF-producing variants such as SfNic-B [27,31].

The contrasting differences in the structural composition and phenotypic traits of the variants present in the SfMNPV-NIC isolate make them an interesting model to study the sociovirology of alphabaculoviruses. However, to better comprehend how host–variant and variant–variant interactions impact the transmission and replication dynamics of variant populations, a variant-specific virus quantification method is essential. In the present study, four genotypic variants were selected that differed in their phenotypic traits and in their prevalence in the SfMNPV-NIC isolate. The aim of the study was therefore to develop an accurate and reproducible method to quantify these four SfMNPV-NIC variants using real-time quantitative PCR (qPCR).

## 2. Materials and Methods

### 2.1. Viral Amplification and DNA Isolation

Variants SfNic-A, SfNic-B, SfNic-C and SfNic-E were originally grown in larvae of the fall armyworm, *S. frugiperda*, at the Universidad Pública de Navarra, Spain [27]. OB suspensions were each amplified in *S. frugiperda* fourth instars by oral inoculation or by injection of ODVs in the case of SfNic-C (that alone is not infectious per os).

For the present study, insects were obtained from a laboratory colony of *S. frugiperda* originating from maize fields in Veracruz State, Mexico (19.43745 N, −96.37787 W). The colony was maintained on a semi-synthetic diet adapted from Mihm [32] (Supplementary Table S1) at 26 ± 1 °C, 70 ± 10% RH, a 14:8 h L:D photoperiod. The colony was known to be free from sublethal SfMNPV infection [33]. Following death, two virus-killed larvae of each genotype were macerated in 1 mL of ultrapure sterile water (GenPure xCAD Plus, Barnstead Water Purification Systems, Thermo Fisher Scientific, Waltham, MA, USA), filtered through an 80 μm steel mesh, and the resulting suspension was centrifuged at 400× *g* for 6 min to sediment insect debris. A 400 μL volume of the resulting supernatant was passed through a 40% (*v*/*v*) glycerol cushion by centrifugation at 5900× *g* for 10 min. The resulting OB pellet was resuspended in 500 μL of ultrapure water, counted in triplicate in an improved Neubauer chamber, and diluted to a concentration of 1 × 10^8^ OBs/mL.

To obtain viral genomic DNA, a 200 μL volume of each OB suspension was mixed with 100 μL of 3× DAS alkaline buffer (0.51 M NaCl, 0.3 M Na_2_CO_3_, 30 mM EDTA, pH 8) and was incubated at 40 °C for 30 min to release the ODVs. Undissolved OBs were removed by centrifugation at 4000× *g* for 10 min. The supernatant was diluted with 500 μL of ultrapure water and centrifuged at 16,000× *g* for 15 min to pellet the ODVs. The ODVs were resuspended in 200 μL of ultrapure water and viral DNA was extracted using the DNAeasy Blood and Tissue Kit (Qiagen, Hilden, Germany) following the manufacturer’s protocol.

### 2.2. Primer Design

Genotype-specific primers were designed based on the published genome of the SfMNPV-NIC variant B (NC_009011.2, NCBI), and sequencing data from the auxiliary gene-rich and structurally variable region in the isolate [34] (Supplemental Figure S1). Primers for SfNic-A, SfNic-C and SfNic-E were previously designed in our laboratories based on the presence or absence of particular genes in the deletion region of each variant (Figure S1). Polyhedrin primers were designed using the reported *polyhedrin* gene sequence (GenBank ID: 5176004). The Primer3Plus tool [35] was used to design qPCR primers with the following parameters: qPCR setting, primer size from 20 to 25 bp, melting temperature of 58–64 °C, and product sizes from 100 to 150 bp. All primer pairs were checked for primer dimer formation and self-complementarity using the ThermoFisher Primer Analyzer tool [36]. Only primer pairs that were not predicted to form dimers, secondary structures, or self-complementarity were selected (Table 1).

### 2.3. Preparation of Standard Templates for qPCR Calibration

The amplicon DNA fragment of each target was initially cloned into a plasmid. For this, each target was amplified by preparing qPCR reactions with 5 μL of iQ SybrGreen 2x Mastermix (Biorad, Hercules, CA, USA), 2.4 μL ultrapure water and 0.5 μM of each primer pair (Table 1) and 1 μL of the DNA template. The cycling conditions consisted of 3 min at 95 °C, followed by 40 cycles of 94 °C for 30 s, 60 °C for 30 s, and 72 °C for 1 min. Each PCR product was cloned using the CloneJet PCR Cloning Kit (Thermo Fisher Scientific, Waltham, MA, USA) and transformed into chemically competent *E. coli* DH5α cells by heat-shock treatment according to standard protocols [37]. Recombinant pJet1.2 plasmids were confirmed by colony PCR using the provided pJET1.2 primers and Sanger sequencing (Macrogen, Seoul, Republic of Korea). The inserted sequences were confirmed to be 100% identical to the published SfNic-B sequence (NC_009011.2, NCBI) or unpublished partial genome sequences from each of the other variants (SfNic-A, SfNic-C and SfNic-E) [38].

Confirmed recombinant plasmids from each insert were used to generate amplicons as standards to construct calibration curves. To this end, PCR reactions were prepared with 12.5 μL GoTaq Mastermix (Promega, Madison, WI, USA), 9.9 μL ultrapure water, 0.5 μM of each pJet1.2 sequencing primer, and 40 ng of pDNA. The cycling program consisted of 3 min at 95 °C, followed by 35 cycles of 94 °C for 30 s, 60 °C for 30 s, and 72 °C for 1 min. PCR products were purified using Wizard^®^ SV Gel and the PCR Clean-Up System (Promega, Madison, WI, USA) and then quantified using a NanoDrop 3000c (Thermo Fisher Scientific, Waltham, MA, USA). The amplicon copy number of each template was calculated using the ThermoFisher DNA Copy Number Calculator [39], using the reference size of the insert plus the pJET overhangs of the primer sequences.

### 2.4. qPCR Calibration Curves

To produce the standard curves for each target (SfNic-A, SfNic-B, SfNic-C, SfNic-E, and *polyhedrin*), PCR reactions were prepared as follows: 5 μL of iQ SybrGreen 2× MasterMix (Biorad, Hercules, CA, USA), 2.4 μL ultrapure water, 0.5 μM of each primer, and 1 μL of the corresponding template dilution starting from 1 × 10^8^ to 1 × 10^1^ copies prepared as 1:10 serial dilutions. Reactions were amplified in 96-well polypropylene plates (Axygen, Corning, NY, USA) and sealed with Microseal adhesive film (Biorad, Hercules, CA, USA). Ultrapure water was used as a negative control. The cycling program consisted of 3 min at 95 °C, followed by 40 cycles of 30 s at 95 °C, 30 s at 60 °C and 1 min at 72 °C using a Stratagene Mx3005p qPCR System (Agilent, Santa Clara, CA, USA). The template samples were plated in triplicate. Each standard curve assay was assessed in three independent experiments.

The R^2^ value, the y-intercept, and reaction efficiency were calculated in the MxPro software v. 4.10 (Agilent, Santa Clara, CA, USA). The dynamic range and limits of detection were determined based on the three independent replicates of the assays for each target. Reproducibility and repeatability of the assay were calculated by estimating intra-assay variation coefficients within technical replicates and inter-assay coefficients of variation (CVs) using experimental replicates corresponding to each of the targets of interest using the equation CV = [standard deviation/mean] × 100% [40,41]. A one-way analysis of variance (ANOVA) was used to determine differences among the intra-assay CV values for all targets covering the complete dynamic range.

### 2.5. Specificity of Variant Primers

The specificity of each primer pair was assessed through cross-validation. The qPCR reactions were prepared with 5 μL of iQ SybrGreen 2× MasterMix, 2.4 μL ultrapure water, and 0.5 μM of each genotype specific primer pair. A 1 ng sample of each variant DNA (1 ng/μL) was tested against each of the primer pairs. Ultrapure water was used as a negative control. Cycling conditions were programmed as described in Section 2.4, and each reaction was performed in triplicate. Specificity was calculated as a percentage, considering the copy number determined for the expected target as 100% and subtracting the percentage of copies for off-target amplification.

### 2.6. Specificity of Primers against a Heterologous Virus (SeMNPV)

A sample of Spodoptera exigua multiple nucleopolyhedrovirus (SeMNPV) was obtained from frozen (−80 °C) material remaining from a previous study [42]. Viral DNA was extracted from a semi-pure suspension of OBs, as described in Section 2.1, and was used to test the specificity of the *polyhedrin* gene and SfMNPV-NIC variant primer pairs. A 1 ng sample (1 ng/μL) of SeMNPV genomic DNA or the expected target for each primer pair (SfNic-A, -B, -C and -E) was used in each reaction. Ultrapure water was used as a negative control. The qPCR reactions and cycling conditions were prepared as described in Section 2.4. Each reaction was performed in triplicate. As before, specificity was calculated by considering the copy number determined for the expected target as 100% and subtracting the percentage of copy numbers for off targets. Melt curves for SfNic-B and the *polyhedrin* gene were performed with 3 min at 95 °C, 1 min at 60 °C and with 2 °C increments up to 94 °C. The resulting curves were calculated using MxPro software, v. 4.10.

### 2.7. Quantification of Genotype-Specific Amplicons in a Mixture

The standard amplicon suspensions were diluted to produce a mixture with 1.5 × 10^4^ copies/μL of each of the four genotypic variant amplicons. Then, the amplicons of each genotypic variant were quantified in the mixture using 1 μL of the suspension as template as previously described in Section 2.4. Each reaction was performed in triplicate and the experiment was performed three times. The relative abundance of each amplicon was calculated using the sum of the determined copy number for each genotypic variant as 100%.

### 2.8. Genotypic Variant Quantification in BV Mixtures

Groups of 10 recently molted fourth instar larvae were inoculated with each variant. For SfNic-A, SfNic-B and SfNic-E, inoculation was performed using the droplet feeding method with a concentration of 1 × 10^7^ OBs/mL. For SfNic-C that is not perorally infectious, an ODV inoculum was prepared from a suspension of 1 × 10^7^ OBs/mL as described in Section 2.1 and finally resuspended in 50 μL of ultrapure water. A 5 μL volume of the SfNic-C ODV suspension was injected into each larva using a microinjector (Burkard, Rickmansworth, UK) fitted with a 1 mL syringe. Inoculated larvae were incubated for 72 h at 27 °C with semi-synthetic diet.

For each of the variants, after 72 h, hemolymph was sampled using the proleg excision method to obtain BVs [43]. Volumes of 10–30 µL of hemolymph were obtained from each larva and were pooled in a microcentrifuge tube containing 200 µL of ice-cold 10 mM L-cysteine to avoid melanization. A 200 µL volume of collected hemolymph in cysteine solution was used directly for viral DNA isolation using the DNAeasy Blood and Tissue Kit (Qiagen, Hilden, Germany). The resulting DNA was suspended in 40 µL of ultrapure water and was quantified using a BioSpecNano (Shimadzu, Kyoto, Japan) and used for qPCR as described in Section 2.4. The genome number was calculated with reference to the constructed standard curves.

The samples of BVs in hemolymph were diluted to prepare mixtures of 2000 copies/µL of each of the four variants in a final volume of 200 µL. This four-variant BV mixture was subjected to DNA isolation and qPCR quantification as described in Section 2.4 to measure the quantity of each of the genotypes in the mixture with a predetermined equimolar composition. Additional reactions were performed using the *polyhedrin* primers for reference in order to verify the total number of viral genomes in the sample (as all variants carried a copy of the *polh* gene). The number of genome copies in each sample was calculated with reference to the constructed quantification curves. This experiment was performed three times in independent assays.

### 2.9. Genotypic Variant Quantification in Mixtures of DNA Extracted from ODVs

An additional validation experiment was performed to ensure that the quantification technique was not affected by the quality of DNA obtained following alkaline lysis of OBs and the use of SDS and proteinase K to release genomic DNA from ODVs. For this, independent samples of 1 × 10^8^ OBs/mL of each variant were subjected to DAS alkaline lysis and SDS + proteinase K treatment at 40 °C, followed by DNA isolation using the DNAeasy Blood and Tissue Kit (QIAGEN, Hilden, Germany), as described in Section 2.1. The DNA concentration of the samples was measured using a BioSpecNano (Shimadzu, Kyoto, Japan) and genome copy number was determined by qPCR amplification using the *polyhedrin* primers and 2 ng of each DNA preparation, as described in Section 2.8. An equimolar mixture of the ODV DNA samples comprising 5 × 10^5^ copies/µL of each variant was prepared and the relative frequencies of each of the genotypic variants and total *polyhedrin* were quantified by qPCR using the constructed standard curves (Section 2.4). This experiment was performed three times in independent assays.

## 3. Results

### 3.1. qPCR Characteristics

A qPCR method was standardized to quantify four individual genotypic variants (SfNic-A, -B, -C, and -E) of the SfMNPV-NIC isolate, along with the *polyhedrin* gene (present in all genotypes) as a reference gene to quantify the overall presence of SfMNPV genomes (Table 2). The efficiency of all amplifications ranged from 97.2% to 100.9% while the dynamic range was between 1 × 10^8^ and 10^1^ copies in all six constructed curves. Mean Cq values increased steadily with increasing template concentration for all variants and the reference genes (Supplementary Table S2).

**Figure 1:**
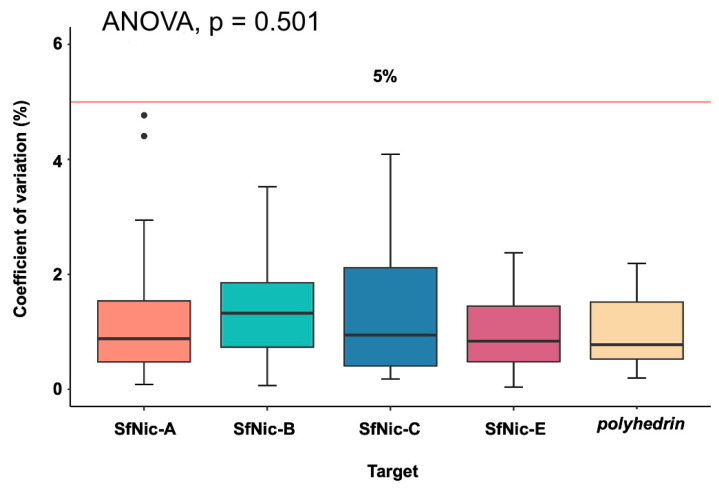
Median coefficients of variation (%) for each genotypic variant and the *polyhedrin* reference gene of SfMNPV. The horizontal red line indicates the 5% CV value taken as the acceptable limit of variability. CV values were calculated for the three replicates of all concentrations included in the dynamic range. Black dots represent outliers. Vertical whiskers display the range of data values from the minimum to the maximum and the interquartile range represented by the box.

Intra-assay variability (CV) ranged from 0.03% to 4.76%, and inter-assay variability ranged from 0.8% to 3.5%. Both parameters were within acceptable limits (<5%) (Supplementary Tables S2 and S3). Importantly, the inter-assay CV value encompassing the complete dynamic range for each of the variants did not vary significantly among the different variants, or the *polyhedrin* amplification (ANOVA, F = 0.842; d.f. = 4, 115; *p* = 0.501) (Figure 1), which simplifies the direct comparison of Cq values.

### 3.2. Primer Specificity

The genotype-specific primer pairs were tested using equal quantities of viral DNA from the four different variants, including their primary target variant (Table 3). The primer pairs for SfNic-A and SfNic-C exclusively amplified their expected targets (100% specificity). The primer pair for SfNic-E showed strong amplification of the main target and minimal amplification of SfNic-A at quantification cycle Cq 38.2, which was beyond the dynamic range of the quantification curve. The specificity of the SfNic-E primer pair was 99.999%. For the SfNic-B primer pair, amplification was detected across all variants but at very low levels (<1%), resulting in a specificity of 99.7%, which would allow us to account for this off-target amplification in a mixed-variant sample.

The primer pairs were also tested against SeMNPV, which is closely related to SfMNPV within group II of the *Alphabaculovirus* genus [44]. The SfNic-A-, SfNic-C- and SfNic-E-specific primers did not present an amplification signal in the SeMNPV sample. In contrast, *polyhedrin* primers amplified the SeMNPV sample at Cq 32.3, whereas an identical concentration of the primary target SfMNPV-NIC DNA resulted in a Cq value of 22.3, indicating 99.93% specificity. SfNic-B primers amplified the SeMNPV sample; however, this was at Cq 33.8, while at the same sample concentration, the SfNic-B DNA sample had a Cq of 23.6, indicating a specificity of 99.92%. The melting curves for qPCR reactions for *polyhedrin* and SfNic-B primers both showed single peaks in the presence of their designed targets or the SeMNPV template suggesting a single amplicon product resulting from each of these primer pairs (Supplementary Figure S2).

### 3.3. Validation of the Quantification Method in Mixtures of Amplicons, BVs and ODV DNA

To validate the functionality of this method, equimolar mixtures were quantified from (i) amplicon standards of each of the variants, (ii) BVs of the genotypic variants and (iii) genomic DNA extracted from ODVs of each variant.

**Figure 2:**
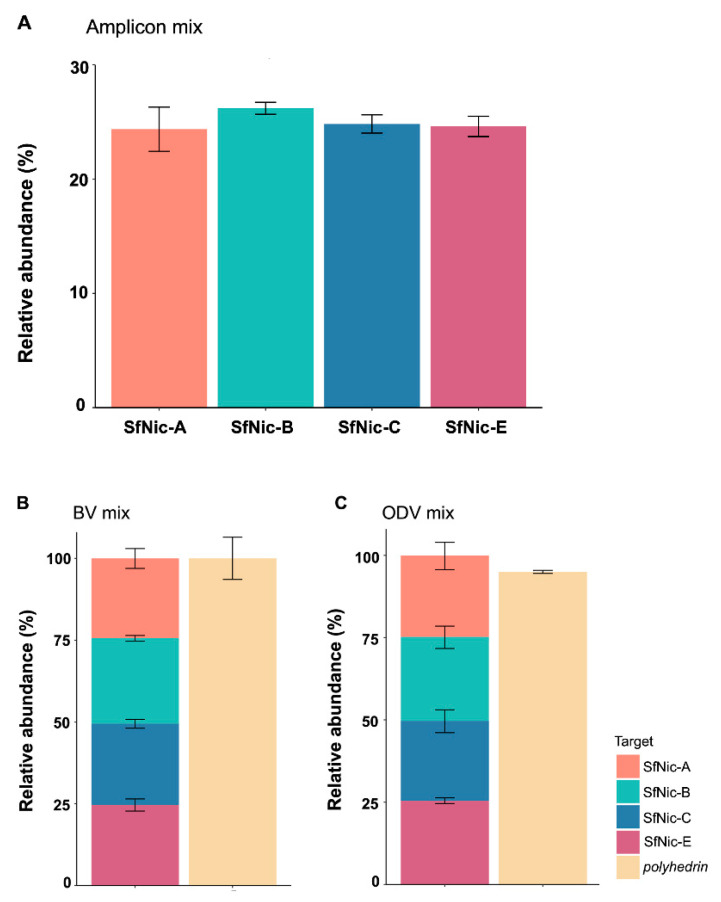
Quantification of equimolar mixtures of (**A**) amplicon standards, (**B**) DNA extracted from BVs in hemolymph and (**C**) DNA extracted from ODVs. The relative abundance of each of the genotypic variants in the mixtures was based on three independent experimental replicates. Error bars indicate SD. The *polyhedrin* gene was quantified to determine the total number of virus genomes present in BV and ODV DNA samples.

The amplicon concentration in the mixtures for each of the genotypic variants across the three replicates ranged from 1.33 × 10^4^ to 1.66 × 10^4^ copies/μL (mean ± SD: 1.49 × 10^4^ ± 0.73 × 10^4^), which compares favorably with the 1.5 × 10^4^ copies/µL samples used to prepare the amplicon mixtures (Figure 2A; Supplementary Table S4). In terms of the mean (±SD) relative abundance of amplicons, the SfNic-A amplicons comprised 24.37 ± 1.93% of the amplicon mixture compared to 26.19 ± 0.52% for SfNic-B, 24.83 ± 0.79% for SfNic-C, and 24.61 ± 0.88% for SfNic-E (Figure 2A).

In the case of the mixtures of variant BVs, these were mixed in equal proportions. BV samples ranged from 3.06 × 10^7^ to 3.86 × 10^3^ genomes/µL (Supplementary Table S5) and were diluted to 2000 copies/µL of each variant in a total volume of 200 µL. The qPCR quantification of this mixture reflected its equimolar composition. Three replicate assays provided consistent results with low variation. The relative abundance of SfNic-A was estimated at 26.37–27.98%; SfNic-B was estimated at 20.36–24.88% of the mixture, SfNic-C was estimated at 18.24–27.81%; and SfNic-E was estimated at 24.21–30.52% (Figure 2B). Quantification of the *polyhedrin* gene provided additional support for the estimates of variant composition. The sum of the number of copies for all variants was 5.14 × 10^4^ in Replicate 1, 4.92 × 10^4^ in Replicate 2 and 4.21 × 10^4^ in Replicate 3. The *polyhedrin* gene yielded copy number estimates of 5.59 × 10^4^ (an 8% difference relative to the estimate based on the sum of all variants), 4.60 × 10^4^ (7% difference) and 4.09 × 10^4^ (3% difference) for Replicates 1, 2 and 3, respectively (Supplementary Table S6).

Finally, the mixtures of ODV variant DNAs extracted from ODVs ranged from 3.97 × 10^5^ to 6.22 × 10^5^ genomes/µL among the different variants (mean ± SD: 5.23 × 10^5^ ± 7.5 × 10^4^; Supplementary Table S7). Averaging across the three independent replicates, the relative abundances of the variants in the mixtures were 24.76% for SfNic-A, 25.55% for SfNic-B, 24.25% for SfNic-C and 25.42% for SfNic-E, reflecting the equimolar structure of the mixture (Figure 2C). The sum of the genome copy number for all the variants in each replicate was 2.14 × 10^6^ in Replicate 1, 2.09 × 10^6^ in Replicate 2 and 2.04 × 10^6^ in Replicate 3, which compares favorably with the *polyhedrin* gene quantification of 2.04 × 10^6^ for Replicate 1 (a 5% difference relative to the estimate based on the sum of all variants), 1.97 × 10^6^ for Replicate 2 (6% difference) and 1.95 × 10^6^ for Replicate 3 (5% difference) (Supplementary Table S7).

## 4. Discussion

Various qPCR methods have been used previously for alphabaculovirus quantification in insect and environmental samples [45,46,47], as well as numerous baculovirus transcriptional studies. The SfMNPV-NIC isolate was previously subjected to qPCR assays using primers targeted at *polyhedrin* [48,49]. However, qPCR methods for quantification of genotypic variants in a nucleopolyhedrovirus isolate are scarce [50]. For this reason, in this study, a qPCR method for accurate quantification of four genotypic variants found in the SfMNPV-NIC isolate was developed and tested. Standardized quantification curves showed high correlation coefficients, desirable efficiency, and a broad dynamic range with a minimum threshold of 10 genome copies/reaction. The assays demonstrated reproducibility and repeatability, as shown by the very low coefficients of variation (median CV 1.0–1.4%, Figure 1). Furthermore, primer specificity ranged from 99.7 to 100%, which ensured that the variants of interest could be accurately quantified in experimental mixtures without interference from other variants or closely related viruses, such as SeMNPV.

Only in the case of the SfNic-B primers was minimal amplification detected in other variant samples. It is important to verify whether such amplification effectively corresponds to off-target amplification or to trace amounts of SfNic-B DNA in the pure samples of the other genotypical variants. However, when tested against the cloned amplicons, none of the primer pairs resulted in amplification. This was confirmed during the quantification of variant amplicon standards in an equimolar preparation, where the composition of the mixture was accurately reflected in the amplification results.

The nucleopolyhedrovirus used in this study was first isolated in Nicaragua and has been extensively characterized for its insecticidal properties and genetic characteristics. The nine genotypical variants present in the SfMNPV-NIC isolate were originally isolated by plaque purification and compared by restriction endonuclease profiles [16,24,25]. Initial efforts to quantify the abundance of each variant in the population involved densitometric analysis of a digoxigenin-labeled restriction fragment (EcoRI 3.1 Kb) and densitometric analysis of semi-quantitative PCR amplifications [27], but these techniques provided markedly different estimates of the prevalence of variants compared to restriction endonuclease analysis of the original plaque picks (Simón et al., 2004). We attribute these discrepancies to marked differences in the propensity of variants to infect and replicate in cell culture conditions and in the semi-quantitative nature of the densitometric techniques employed.

Reassuringly, the total number of variant genome copies quantified by qPCR on BV mixtures deviated only slightly from the total number of viral genome copies quantified by amplification of the *polyhedrin* gene present in all variants. A similar result was obtained using mixtures of DNA extracted from ODVs, indicating that the precision of the qPCR technique was not affected by the extraction process applied to obtain gDNA from ODVs.

Variation in qPCR assays can result from several sources such as inconsistencies in the pipetting technique. However, the baseline correction algorithms present in Agilent MxPro software automatically correct for most variation due to aliquoting errors without the need for reference dye normalization. In addition, when using purified amplicons as template standards, it is important to confirm the integrity of these over time to ensure accurate quantification [51]. Finally, the quality of the DNA sample can influence reaction efficiency and thus affect the overall quantification [40]. For this reason, we employed a DNA purification kit based on silica spin columns rather than in-house protocols involving phenol–chloroform treatment, which tended to produce samples of variable quality in preliminary testing. We observed low levels of variation among the BV mixed-variant preparations and among mixtures of DNA from ODVs. For BVs, this likely resulted from variation in DNA quality in the hemolymph samples that can experience varying degrees of melanization during collection and storage. Virus viability and the quality of genomic DNA in occluded ODVs may also vary during storage even under low temperature conditions [52,53,54]. For this reason, all the OB samples used in the present study spent less than three months in storage at −10 °C.

Studies in progress employ this technique to quantify variants in co-occluded mixtures used as inoculum in transmission experiments and to monitor the replication and progeny production in mixed-variant infection [55,56]. Another advantage of the current methodology is that it allows for the direct quantification of targets from any DNA sample, including OB suspensions, infected host tissues and potentially environmental samples such as plants and soil. Moreover, it is also possible to extend the methodology to other genotypic variants of this isolate and other alphabaculoviruses. This methodology can aid in the development of custom viral insecticides that take advantage of the interactions between specific variants to produce biological insecticides with desirable pest control properties.

## Figures and Tables

**Table 1:** List of primers used to amplify each target.

Target	Primer Sequence (5′-3′)	Tm (°C)	Amplicon Size (bp)
SfNic-A	AFw 5′-TCGAGCGTTCGTAACATTGTG-3′	60.2	113
	ARv 5′-GGCCAAATTCAAAACGGAAA-3′	56.8	
SfNic-B	BFw 5′-ACACCACCGAACTGACTTGGAACGA-3′	59.9	103
	BRv 5′-GTTCGTCGGCAGTACATGAATC-3′	59.7	
SfNic-C	CFw 5′-GCCGCGTTTAGTAACAGCAAA-3′	60.4	150
	CRv 5′-TGATTTTCTTCCGTTCTCTGACAC-3′	60.2	
SfNic-E	EFw 5′-TCTTGGTCATGTCCGCAAAA-3′	57.1	122
	ERv 5′-CGCGCTCGATCGTGAGTAT-3′	58.6	
*polyhedrin*	polhFw 5′-GCCCGTGTACGTAGGAAACA-3′	59.3	110
	polhRv 5′-ACTCTTCGAAGGAGTGCGTG-3′	59.1	

**Table 2:** qPCR amplification characteristics determined by MxPro. Mean values ± SD were determined from three independent replicates of each assay.

Target	R^2^	Slope	y-Intercept	Efficiency (%)
SfNic-A	0.957 ± 0.040	−3.354 ± 0.040	40.74 ± 0.53	98.9 ± 2.1
SfNic-B	0.993 ± 0.030	−3.334 ± 0.003	38.86 ± 0.70	99.4 ± 0.2
SfNic-C	0.989 ± 0.004	−3.479 ± 0.200	39.78 ± 0.37	97.2 ± 2.5
SfNic-E	0.993 ± 0.005	−3.335 ± 0.700	39.19 ± 0.21	98.5 ± 1.4
*polyhedrin*	0.998 ± 0.003	−3.269± 0.050	35.92 ± 0.30	100.9 ± 1.1

**Table 3:** Genotype-specific primer specificity (%). Each primer amplification was compared to the constructed quantification curves for each target.

Specificity (%)				
Sample	SfNic-A	SfNic-B	SfNic-C	SfNic-E
SfNic-A	100	0.05	–	0.0015
SfNic-B	–	99.7	–	–
SfNic-C	–	0.2	100	–
SfNic-E	–	0.05	–	99.999

## Data Availability

The original contributions presented in the study are included in the supplementary tables and figures. Further inquiries can be directed to the corresponding author.

## Supplementary Materials

The following supporting information can be downloaded at: https://www.mdpi.com/article/10.3390/v16060881/s1, Table S1: Semi-synthetic diet for *Spodoptera frugiperda* larvae; Table S2: Intra-assay variability for all assays; Table S3: Inter-assay variability for all targets; Table S4: qPCR quantification of variant-specific amplicon standards in a mixture; Table S5: BV count of hemolymph samples taken at 72 h post inoculation; Table S6: qPCR quantification of SfMNPV-NIC genotypes in a mixture of BVs; Table S7: qPCR quantification of SfMNPV-NIC variants in a mixture of genomic DNAs extracted from ODVs of each variant. Figure S1: Schematic of the position of forward (fw) and reverse (rv) primer targets for the amplification of complete variant SfNic-B and the deletion variants SfNic-A, SfNic-C and SfNic-E; Figure S2: Melting curves for *polyhedrin* and SfNic-B amplicons.
