# Supplementary material for: A qPCR Assay for the Quantification of Selected Genotypic Variants of Spodoptera frugiperda Multiple Nucleopolyhedrovirus (Baculoviridae)
Source: Viruses. 2024 May 30;16(6):881. doi: 10.3390/v16060881 (PMC11209410; doi:10.3390/v16060881)
Supplement: Supplementary file 1 [file viruses-16-00881-s001.zip › viruses-2993968-supplementary.pdf]

SUPPLEMENTARY MATERIAL: A qPCR assay for the quantification of selected genotypic variants of Spodoptera frugiperda multiple nucleopolyhedrovirus (Baculoviridae)

Molina-Ruiz et al.

Table S1: Semi-synthetic diet for *Spodoptera frugiperda* larvae

To prepare ~1 L of diet:

Ingredient	Quantity
Soybean flour	81 g
Wheatgerm	32 g
Brewer's yeast	25 g
Sucrose	13 g
Carrageenan	28 g
Potable water	1000 mL
*Potassium sorbate	1.0 g
*Methyl paraben	1.6 g
*Ascorbic acid	4.3 g
*Multivitamin mixture (Vitafort-A, Parfarm, Mexico City)	1.0 g
*Oxytetracycline chlorhydrate	130 mg
*25% acetic acid solution	12 mL
*15% choline chloride solution	7.3 mL
**10% formaldehyde solution	4.4 mL
***Mixture of salts	8.0 g

* Compounds mixed in a 50 mL volume of water and added when the diet is 70 °C and then cooled rapidly.

** Formaldehyde is not included in diet destined for bioassays or other virus studies.

*** Simplified mixture of salts comprising 21 g $CaCO_3$; 1.47 g $Fe_2(SO_4)_3$; 9 g $MgCl.nH_2O$; 12 g KCl; 31 g K_2HPO_4 (anhydrous); 10.5 g NaCl; 14.9 g $Ca_3(PO_4)_2$

Diet recipe adapted from Mihm (1984) Técnicas Eficientes para la Crianza Masiva e Infestacion de Insectos, en la Selección de las Plantas Hospedantes para Resistencia al Gusano Cogollero, *Spodoptera frugiperda*. Centro Internacional de Mejoramiento de Maiz y Trigo (CIMMYT): El Batán, Mexico, 1984; pp. 16.

Table S2. Intra-assay variability for all assays. Mean Cq values from three independent replicates in each assay. The means, SD and coefficient of variation (%) values were calculated for each sample based on three technical replicates present in each plate.

polyhedrin	**Replicate 1**			**Replicate 2**			**Replicate 3**		
**copies**	**Mean Cq**	**SD**	**CV (%)**	**Mean Cq**	**SD**	**CV (%)**	**Mean Cq**	**SD**	**CV (%)**
**10^8**	9.30	0.10	1.10	9.78	0.19	1.98	9.56	0.15	1.54
**10^7**	12.68	0.07	0.53	12.98	0.10	0.76	12.69	0.08	0.61
**10^6**	16.20	0.05	0.29	16.40	0.11	0.67	16.42	0.09	0.52
**10^5**	19.29	0.04	0.20	19.82	0.30	1.50	19.69	0.06	0.29
**10^4**	23.10	0.25	1.06	22.92	0.12	0.52	24.38	0.06	0.26
**10^3**	26.17	0.21	0.79	26.67	0.40	1.51	26.38	0.15	0.55
**10^2**	29.25	0.35	1.20	29.94	0.19	0.63	29.60	0.65	2.19
**10^1**	32.20	0.55	1.72	32.97	0.67	2.04	32.00	0.52	1.63
**SfNic-A**	**Replicate 1**			**Replicate 2**			**Replicate 3**		
**copies**	**Mean Cq**	**SD**	**CV (%)**	**Mean Cq**	**SD**	**CV (%)**	**Mean Cq**	**SD**	**CV (%)**
**10^8**	13.07	0.37	2.81	12.86	0.61	4.77	12.53	0.08	0.66
**10^7**	17.07	0.17	1.01	17.34	0.14	0.81	17.30	0.76	4.40
**10^6**	21.18	0.19	0.90	21.26	0.15	0.68	21.44	0.02	0.10
**10^5**	24.93	0.13	0.52	24.96	0.07	0.29	24.55	0.02	0.08
**10^4**	28.64	0.09	0.33	28.06	0.49	1.75	29.30	0.29	1.01
**10^3**	32.35	0.27	0.84	32.39	0.49	1.52	32.46	0.28	0.86
**10^2**	37.22	0.59	1.59	36.26	0.43	1.18	37.05	0.47	1.28
**10^1**	39.44	1.16	2.94	38.88	0.10	0.26	39.13	0.12	0.30
**SfNic-B**	**Replicate 1**			**Replicate 2**			**Replicate 3**		
**copies**	**Mean Cq**	**SD**	**CV (%)**	**Mean Cq**	**SD**	**CV (%)**	**Mean Cq**	**SD**	**CV (%)**
**10^8**	12.64	0.23	1.79	12.16	0.08	0.66	12.21	0.34	2.80
**10^7**	16.03	0.16	1.01	15.37	0.24	1.54	16.13	0.50	3.09
**10^6**	19.64	0.42	2.15	18.58	0.38	2.03	18.29	0.52	2.82
**10^5**	22.62	0.37	1.64	21.40	0.10	0.48	21.48	0.29	1.34
**10^4**	26.22	0.09	0.35	24.85	0.06	0.24	26.01	0.34	1.30
**10^3**	29.61	0.17	0.57	28.27	0.02	0.06	29.35	1.03	3.52
**10^2**	33.27	0.47	1.42	31.66	0.28	0.88	32.15	0.49	1.51
**10^1**	35.00	0.41	1.16	34.53	0.40	1.16	35.51	0.27	0.76
**SfNic-C**	**Replicate 1**			**Replicate 2**			**Replicate 3**		
**copies**	**Mean Cq**	**SD**	**CV (%)**	**Mean Cq**	**SD**	**CV (%)**	**Mean Cq**	**SD**	**CV (%)**

**10^8**	12.83	0.03	0.29	12.3	0.25	2.09	13.39	0.37	2.82
**10^7**	16.5	0.07	0.48	16.08	0.18	1.17	17.26	0.15	0.91
**10^6**	19.86	0.05	0.27	19.79	0.06	0.34	20.81	0.2	0.98
**10^5**	23.24	0.04	0.17	23.82	0.1	0.43	24.5	0.23	0.96
**10^4**	26.67	0.15	0.56	27.78	0.26	0.96	28.17	0.1	0.368
**10^3**	30.26	0.11	0.39	31.22	0.68	2.2	32.09	0.23	0.74
**10^2**	33.94	0.82	2.43	33.59	1.37	4.08	34.99	0.92	2.63
**10^1**	35.55	0.76	2.15	36.44	0.14	0.41	36.43	0.73	2.02
**SfNic- E**	**Replicate 1**			**Replicate 2**			**Replicate 3**		
**copies**	**Mean Cq**	**SD**	**CV (%)**	**Mean Cq**	**SD**	**CV (%)**	**Mean Cq**	**SD**	**CV (%)**
**10^8**	11.67	0.15	1.33	12.44	0.23	1.92	12.59	0.004	0.03
**10^7**	15.29	0.19	1.25	15.94	0.02	0.18	15.84	0.13	0.87
**10^6**	18.46	0.07	0.42	19.06	0.06	0.35	19.13	0.12	0.6
**10^5**	22.1	0.12	0.58	22.64	0.11	0.49	22.54	0.24	1.08
**10^4**	25.42	0.23	0.93	25.95	0.1	0.39	26.02	0.2	0.79
**10^3**	28.92	0.07	0.27	29.2	0.15	0.52	29.38	0.22	0.75
**10^2**	32.7	0.72	2.22	33.47	0.79	2.37	33.12	0.58	1.78
**10^1**	34.87	0.79	2.26	34.92	0.44	1.26	35.5	0.81	2.3

Table S3: Inter-assay variability for all targets. Mean Cq values from three separate assays for each target. The coefficient of variation was calculated from the corresponding assay replicates.

polyhedrin			
**copies**	**Mean Cq**	**SD**	**CV(%)**
**10^8**	9.54	0.25	2.60
**10^7**	12.78	0.16	1.26
**10^6**	16.34	0.13	0.80
**10^5**	19.60	0.29	1.47
**10^4**	22.97	0.19	0.81
**10^3**	26.41	0.34	1.29
**10^2**	28.67	0.52	1.82
**10^1**	32.39	0.72	2.23
**SfNic-A**			
**copies**	**Mean Cq**	**SD**	**CV(%)**
**10^8**	12.82	0.47	3.68
**10^7**	17.24	0.47	2.74
**10^6**	21.29	0.18	0.83
**10^5**	24.81	0.21	0.83
**10^4**	28.33	0.41	1.45
**10^3**	32.40	0.36	1.12
**10^2**	35.31	0.66	1.86
**10^1**	39.15	0.71	1.82
**SfNic-B**			
**copies**	**Mean Cq**	**SD**	**CV(%)**
**10^8**	12.34	0.32	2.61
**10^7**	15.84	0.47	2.98
**10^6**	18.84	0.73	3.87
**10^5**	21.83	0.62	2.84
**10^4**	25.35	0.65	2.57
**10^3**	29.07	0.84	2.89
**10^2**	31.29	0.80	2.55
10^1	35.02	0.54	1.54
**SfNic-C**			
**copies**	**Mean Cq**	**SD**	**CV(%)**
**10^8**	12.67	0.53	4.25
**10^7**	16.45	0.52	3.20
**10^6**	19.88	0.64	3.23
**10^5**	23.53	0.73	3.10
**10^4**	27.44	0.72	2.65

**10^3**	30.79	1.01	3.29
**10^2**	33.82	1.24	3.68
**10^1**	36.02	1.16	3.24
**SfNic-E**			
**copies**	**Mean Cq**	**SD**	**CV(%)**
**10^8**	12.23	0.43	3.57
**10^7**	15.69	0.31	2.02
**10^6**	18.88	0.31	1.67
**10^5**	22.42	0.29	1.30
**10^4**	25.79	0.33	1.28
**10^3**	29.17	0.24	0.85
**10^2**	33.1	0.77	2.34
**10^1**	35.08	0.73	2.10

Table S4: qPCR quantification of variant specific amplicon standards in a mixture. Mean Cq values were calculated from three technical replicates within each replicate. The quantification of copy number (± SD) was performed using the constructed quantification curves.

	Target	Mean Cq	Copies	SD
**Replicate 1**	SfNic-A	26.91	1.33E+04	0.73
	SfNic-B	24.93	1.50E+04	0.42
	SfNic-C	25.34	1.41E+04	0.90
	SfNic-E	25.40	1.36E+04	0.26
**Replicate 2**	SfNic-A	26.87	1.37E+04	0.22
	SfNic-B	24.84	1.60E+04	0.12
	SfNic-C	25.19	1.57E+04	0.19
	SfNic-E	25.18	1.58E+04	0.31
**Replicate 3**	SfNic-A	26.59	1.66E+04	0.12
	SfNic-B	24.87	1.57E+04	0.09
	SfNic-C	25.29	1.46E+04	0.26
	SfNic-E	25.30	1.46E+04	0.91

Table S5: BV count of hemolymph samples taken at 72 h post-inoculation. Mean Cq values were calculated from three technical replicates. The copy number corresponds to the calculation of constructed standard curves. BV concentrations (BVs/µL) correspond to the qPCR quantification result, multiplied by the gDNA elution volume, and divided by the sample volume utilized in the gDNA isolation.

**SfNic-A**	**Mean Cq**	**copies**	**SD**	**BVs/µL**
Replicate 1	13.31	1.53E+08	0.27	3.06E+07
Replicate 2	15.40	3.58E+07	0.05	7.17E+06
Replicate 3	24.54	6.85E+04	0.26	1.37E+04
**SfNic-B**				
Replicate 1	18.55	1.24E+06	0.17	2.48E+05
Replicate 2	20.48	3.72E+05	1.00	7.44E+04
Replicate 3	14.74	1.74E+07	0.25	3.48E+06
**SfNic-C**				
Replicate 1	15.00	1.34E+07	0.29	2.68E+06
Replicate 2	24.88	1.93E+04	0.21	3.86E+03
Replicate 3	15.89	7.39E+06	0.18	1.48E+06
**SfNic-E**				
Replicate 1	16.19	7.94E+06	0.18	1.59E+06
Replicate 2	22.23	1.27E+05	0.51	2.55E+04
Replicate 3	13.44	5.38E+07	0.36	1.08E+07

Table S6: qPCR quantification of SfMNPV-NIC genotypes in a mixture of BVs. Mean Cq values were calculated from three technical replicates. The quantification of copy number was performed using the constructed quantification curves.

	Target	Mean Cq	Copies	SD
**Replicate 1**	SfNic-A	26.80	1.44E+04	0.13
	SfNic-B	25.46	1.05E+04	0.19
	SfNic-C	25.58	1.21E+04	0.07
	SfNic-E	25.32	1.44E+04	0.06
	polyhedrin	20.41	5.59E+04	0.14
**Replicate 2**	SfNic-A	26.95	1.30E+04	0.10
	SfNic-B	25.23	1.22E+04	0.24
	SfNic-C	26.03	8.96E+03	0.02
	SfNic-E	25.26	1.50E+04	0.25
	polyhedrin	20.69	4.60E+04	0.24
**Replicate 3**	SfNic-A	27.11	1.16E+04	0.10
	SfNic-B	25.75	8.57E+03	0.38
	SfNic-C	25.63	1.17E+04	0.01
	SfNic-E	25.82	1.02E+04	0.19
	polyhedrin	20.85	4.09E+04	0.10

Supplementary Table S7: qPCR quantification of SfMNPV-NIC variants in a mixture of genomic DNAs extracted from ODVs of each variant. Mean Cq values were calculated from three technical replicates within each of three biological replicates. The quantification of copy number was performed using the constructed quantification curves.

	Target	Mean Cq	Copies	SD
**Replicate 1**	SfNic-A	21.33	6.13E+05	0.60
	SfNic-B	19.74	5.43E+05	0.27
	SfNic-C	20.24	4.14E+05	0.26
	SfNic-E	19.99	5.70E+05	0.40
	polyhedrin	15.29	2.04E+06	0.16
**Replicate 2**	SfNic-A	21.96	3.97E+05	0.60
	SfNic-B	19.54	6.22E+05	0.27
	SfNic-C	19.80	5.55E+05	0.26
	SfNic-E	20.14	5.15E+05	0.40
	polyhedrin	15.34	1.97E+06	0.13
**Replicate 3**	SfNic-A	21.50	5.45E+05	0.60
	SfNic-B	20.05	4.39E+05	0.27
	SfNic-C	19.81	5.49E+05	0.22
	SfNic-E	20.16	5.10E+05	0.40
	polyhedrin	15.36	1.95E+06	0.02

Figure S1

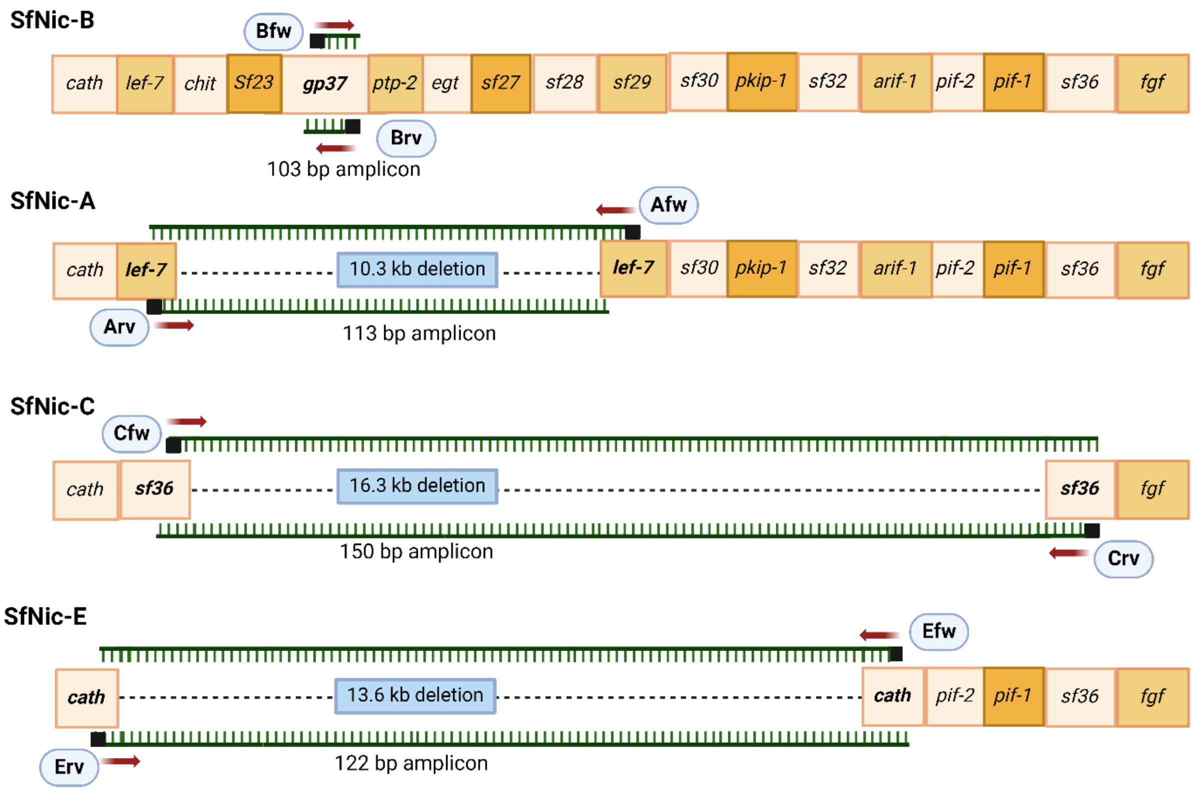

Figure S1. Schematic of the position of forward (fw) and reverse (rv) primer targets for the amplification of complete variant SfNic-B and the deletion variants SfNic-A, SfNic-C and SfNic-E. Target genes for each pair of primers are shown in bold text.

Figure S2

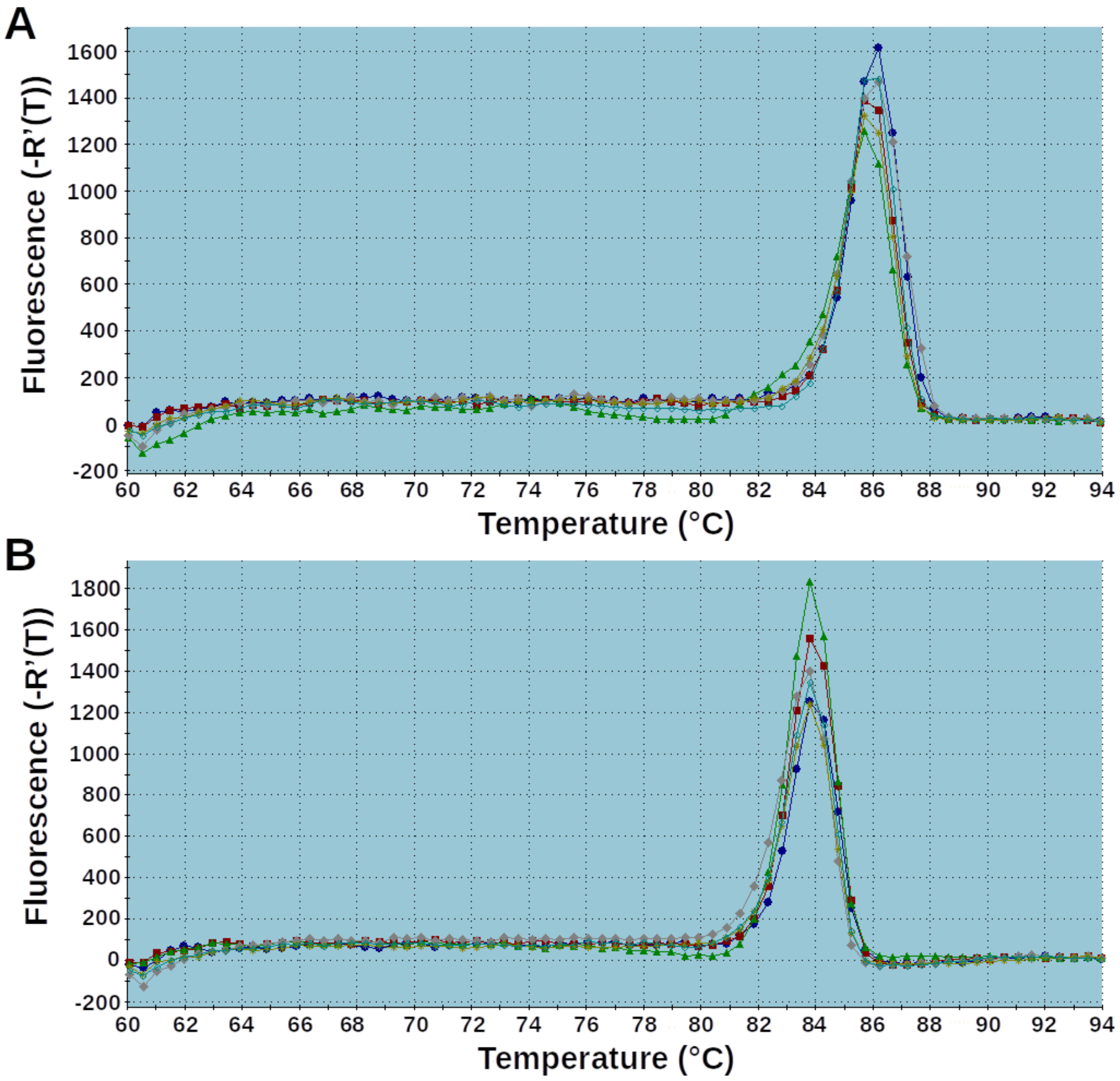

Figure S2. Melting curves for polyhedrin and SfNic-B amplicons. A) Melting curve for *polyhedrin* qPCR reactions. The gDNAs from SfMNPV-NIC and SeMNPV were used as templates. A single peak is observed in the resulting melting curves. B) Melting curve for SfNic-B qPCR reactions. gDNAs from SfNic-B and SeMNPV were used as templates. A single peak is observed in the resulting melting curves.
